# Impacts of a Rapidly Declining Mountain Snowpack on Streamflow Timing in Canada’s Fraser River Basin

**DOI:** 10.1038/srep19299

**Published:** 2016-01-27

**Authors:** Do Hyuk Kang, Huilin Gao, Xiaogang Shi, Siraj ul Islam, Stephen J. Déry

**Affiliations:** 1 Environmental Science and Engineering Program, University of Northern British Columbia, Prince George, British Columbia, Canada; 2 NASA Goddard Space Flight Center, Greenbelt, Maryland, United States of America; 3 Zachry Department of Civil Engineering, Texas A & M University, College Station, Texas, United States of America; 4 CSIRO Land and Water, Canberra, Australian Capital Territory, Australia

## Abstract

With its headwaters in the water towers of the western Cordillera of North America, the Fraser River is one of the continent’s mightiest rivers by annual flows, supplies vital freshwater resources to populous downstream locations, and sustains the world’s largest stocks of sockeye salmon along with four other salmon species. Here we show the Variable Infiltration Capacity (VIC) model’s ability to reproduce accurately observed trends in daily streamflow for the Fraser River’s main stem and six of its major tributaries over 1949-2006 when air temperatures rose by 1.4 °C while annual precipitation amounts remained stable. Rapidly declining mountain snowpacks and earlier melt onsets result in a 10-day advance of the Fraser River’s spring freshet with subsequent reductions in summer flows when up-river salmon migrations occur. Identification of the sub-basins driving the Fraser River’s most significant changes provides a measure of seasonal predictability of future floods or droughts in a changing climate.

The Fraser River Basin (FRB) forms one of the largest and most important watersheds draining the western Cordillera of North America^1^. The FRB spans 240,000 km^2^ of diverse landscapes including 11 different biogeoclimatic zones, from dry interior plateaus to wet, snowy mountains in its headwaters of the northern Rockies, the Columbia and Coast Mountains (Fig. 1). Owing to its rich diversity and abundance of natural resources, the FRB offers a plethora of economic opportunities (e.g., in agriculture, fishing, forestry, mining and hydropower production), provides cultural and societal values, and attracts many tourists and recreationists in its largely undisturbed, scenic landscapes^1^. It also provides extensive terrestrial and aquatic habitat for iconic large mammals and fish species such as grizzly and black bears, mountain caribou, wolves, wolverines, cougars, moose, white sturgeon and five species of salmon. Indeed, the Fraser River and its tributaries sustain the largest migrations of Pacific Ocean sockeye salmon^2^.

There is mounting evidence that climate change has influenced the FRB’s hydrologic regime during the 20th and early 21st centuries, affecting its economic vitality and pristine landscapes^3^,^4^,^5^. Mean annual air temperatures warmed by 1.4 °C between 1949 and 2006 across the FRB while total annual precipitation remained stable despite a significant change in its type from snowfall to rainfall^6^. These changes in the FRB’s climate have impacted the evolution and duration of its seasonal snowpack, resulting in a 19% decline in the contribution of snow to its hydrological regime^6^,^7^,^8^. This suggests the FRB is currently transitioning from a nival to a pluvio-nival hybrid system^6^,^9^,^10^, raising concerns about the impacts of its changing hydrology on terrestrial and aquatic species and their habitats^11^,^12^ as well as on flood and drought occurrences.

While trends in streamflow timing are commonly used as indicators of climate change^13^, identifying the driving factors for observed changes remain quite challenging^14^. Rising air temperatures coupled with changing precipitation amounts and types affect mountain snowpack accumulation and ablation processes that, in turn, lead to modifications in streamflow timing. Large systems, such as the FRB, integrate spatially and temporally complex physical processes that can confound climate change signals and their hydrological impacts. To resolve some of these issues, the application of a semi-distributed, macroscale hydrological model can provide insights on the various agents of change from the grid- to basin-scale. Thus this paper addresses three key research goals: 1) to evaluate the performance of a semi-distributed, macroscale hydrological model in simulating FRB observed streamflow trends at a daily timescale; 2) to quantify the relative contribution and its change over time of the FRB’s main sub-basins to total streamflow on the main-stem lower Fraser River; and 3) to assess the impacts of rising air temperatures, of changes in precipitation types and amounts, and of rapidly declining mountain snowpacks on changes in streamflow timing and amounts across the FRB. To achieve these objectives, both observational and modelling datasets are applied to investigate changes to the hydrologic regime of the FRB from 1949 to 2006.

## Results

The six sub-basins examined in the present study contribute 75.0% (67.9%) of the annual observed (simulated) Fraser River discharge at Hope, British Columbia (BC) with the largest contributions from the Thompson-Nicola (TN), Upper Fraser (UF) and Quesnel (QU) sub-basins (Table 1). Based on observations, the relative contributions of the FRB’s western sub-basins (Stuart (SU), Nautley (NA) and Chilko (CH)) to the annual discharge of the Lower Fraser River at Hope (LF) declined whereas those of the eastern sub-basins have generally increased over the study period. The Variable Infiltration Capacity (VIC) model accurately simulates trends in daily streamflow observed across the FRB, with Nash-Sutcliffe Efficiency (NSE) scores between 0.47 and 0.97, statistically significant correlations and relatively low measures of error (Table 2). Note however the VIC model’s reduced performance over the Stuart, Nautley and Chilko river sub-basins, owing perhaps to deficiencies in the forcing data and the lack of the representation of large, deep lakes, glaciers and land cover changes in the current model’s formulation.

The observed (simulated) daily streamflow for the Fraser River at Hope during April and May exhibits increasing trends with a maximum of 1481 m^3^ (s 58 yrs)^−1^ (2003 m^3^ (s 58 yrs)^−1^) in response to an earlier onset of snowmelt (Fig. 2a). In contrast, the results also show observed (simulated) decreasing trends in streamflow with a minimum of −1780 m^3^ (s 58 yrs)^−1^ (−1495 m^3^ (s 58 yrs)^−1^) but over a prolonged period (June, July and August). Trends in daily streamflow throughout the remainder of the water year are relatively modest in magnitude and are not statistically significant (*p* > 0.05; see Supplementary Fig. 1). Integrating over time the observed and simulated daily streamflow trends reveals the insignificant changes in total annual discharge values, confirming the reported changes are driven by phase shifts as the FRB is transitioning from a nival to a hybrid/pluvial regime.

**Figure 1:**
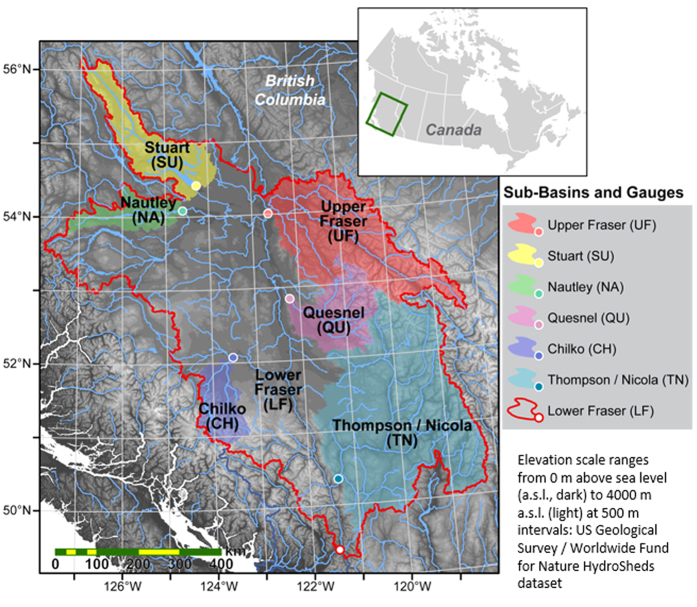
Map of the Fraser River Basin, western Canada showing the six sub-basins of interest including the location of their hydrometric gauges.

**Figure 2:**
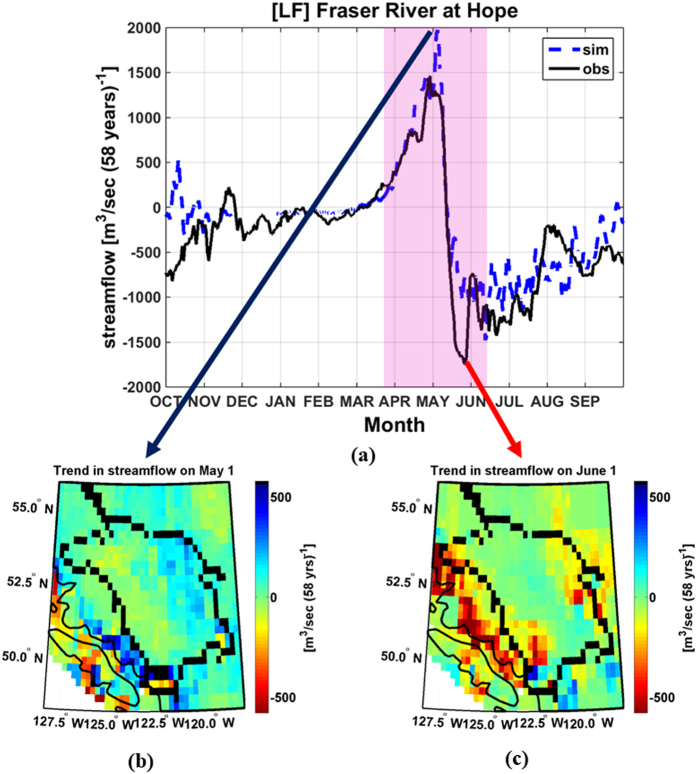
(**a**) Trend in observed (obs) and simulated (sim) daily streamflow for the Fraser River at Hope, BC, water years 1949–2006. Statistically significant trends for the simulated streamflow are shown in the shaded area. Trends in grid-scale simulated daily streamflow across the FRB (**b**) on 1 May and (**c**) on 1 June, water years 1949–2006. Panels (**b**,**c**) were created in Matlab 8.4.0.150421 (R2014b), a software package produced by Mathworks®: http://www.mathworks.com/products/matlab/.

Grid-scale trends in simulated daily streamflow for 1 May (Fig. 2b) and 1 June (Fig. 2c) provide insights on the spatial distribution of these changes. On 1 May, most areas show positive trends with the more amplified values > 200 m^3^ (s 58 yrs)^−1^ found in the lower Coast Mountains, parts of the Upper Fraser, and the windward side of the Canadian Rockies. While the 1 May positive streamflow trends are, in general, spatially coherent, the 1 June negative streamflow trends are isolated to the Coast Mountains and the Canadian Rockies, in the mountainous terrain of the Fraser River.

**Figure 3:**
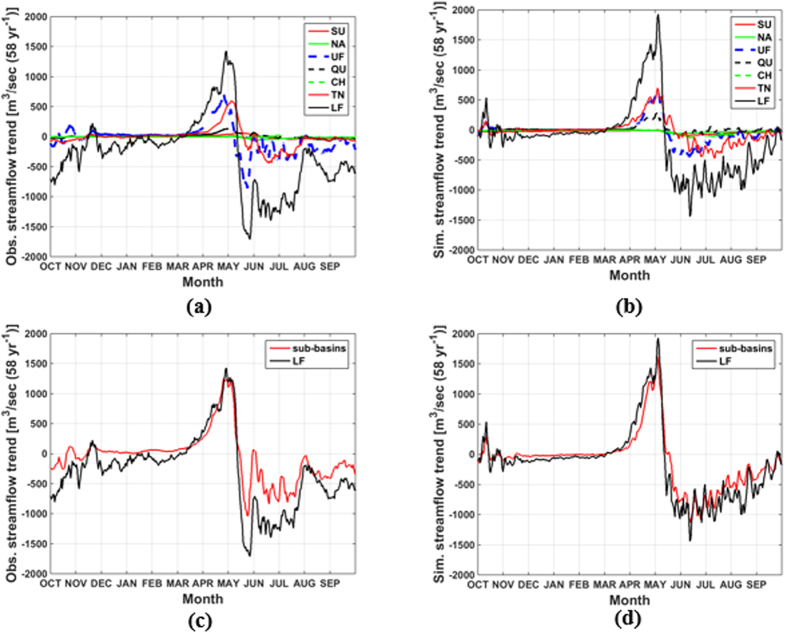
Trends in (a) observed and (b) simulated daily streamflow for all sub-basins of interest (see Table 1) and the Fraser River at Hope (LF), water years 1949–2006. Sum of the trends in (**c**) observed and (**d**) simulated daily streamflow for all sub-basins and comparisons with trends for the Fraser River at Hope, water years 1949–2006.

Given the FRB’s vast area, it is imperative to investigate the contributions of each of its major sub-basins to the observed and simulated trends in daily streamflow. The strong positive trends in daily streamflow in April and May followed by their declining trends in summer are driven mainly by changes in the UF and TN sub-basins (Fig. 3a,b). These two sub-basins have the largest mean annual flows (Table 1). The summations of the time series of daily trends in observed and simulated streamflow for the six major sub-basins match closely the values for the Fraser River at Hope, BC with *r* = 0.94 and 0.96 (both with *p* < 0.05), respectively (Fig. 3c,d). This clearly illustrates the upscaling of streamflow timing changes that occurs from individual sub-basins to the overall larger FRB, and is a feature that the VIC model represents faithfully.

**Figure 4:**
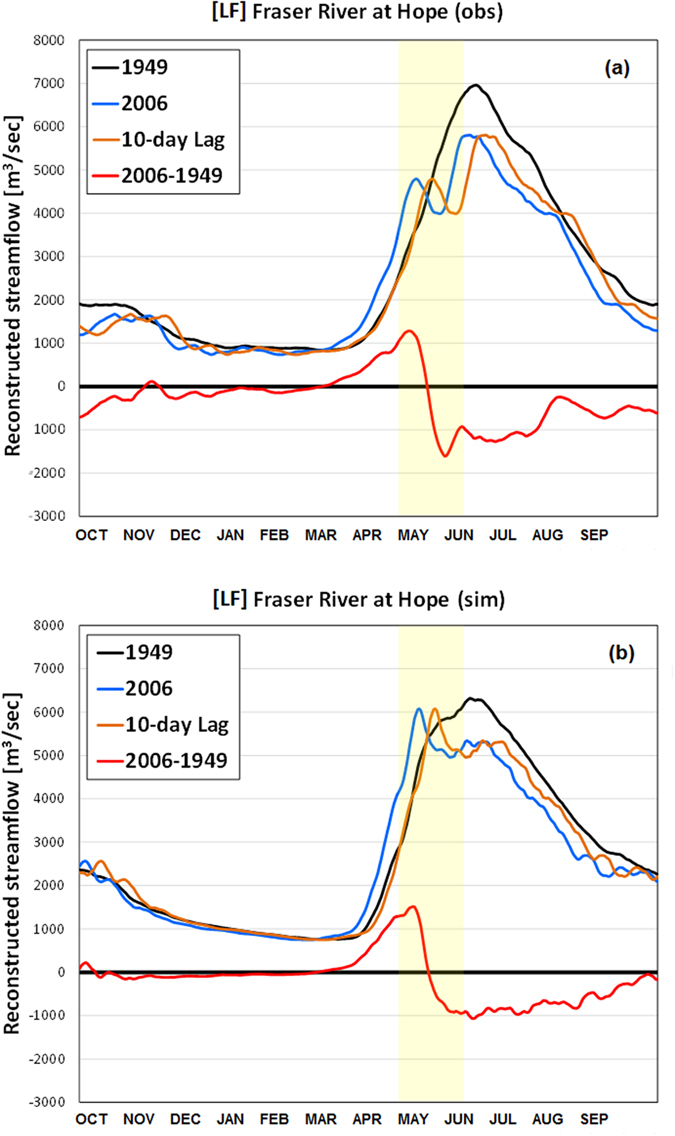
The reconstructed hydrographs for the Fraser River at Hope based on the linear trends in (a) observed (obs) and (b) simulated (sim) 5-day moving averages of daily streamflow and their differences for water years 1949 and 2006. A 10-day lag of the 2006 reconstructed hydrograph is also shown to illustrate the recent advance of the onset of the spring freshet.

Using the slopes determined by the Mann-Kendall Test (MKT) method and median values of the annual daily streamflow as a y-intercept for the study period, the streamflow in a single water year is reconstructed for 1949 and 2006 using observed and simulated streamflow, respectively at Hope, BC (Fig. 4). In 2006, streamflow in April and May reconstructed using the MKT increased by at least 1500 m^3^ s^−1^ (based on both the observed and simulated data) owing to the earlier onset of the spring freshet. Lagging the 2006 reconstructed hydrographs by 10 days relative to the 1949 ones confirm the recent 10-day advances of the onset of the spring freshets for the Fraser River at Hope (see also Supplementary Table 1). In contrast, the duration of diminishing streamflow extends from June to August, suggesting declining summer flows across the FRB. The declines persist during the recession to lower flows in autumn just when the salmon are migrating up the Fraser River. Despite the phase shift of daily streamflow values, the total annual streamflow does not change significantly.

Climatic trends in air temperature, precipitation (including snowfall), snow water equivalent (SWE), runoff and evapotranspiration explain the streamflow trends for water years 1949–2006 (Supplementary Fig. 2). Air temperatures warmed mainly during winter (up to 5 °C in January) as snowfall declined by up to 35 mm month^−1^ with a seasonal deficit of 103 mm. In turn, SWE declined by 105 mm by the time of its peak accumulation. The combined effects of the warmer air temperatures, reduced snowfall and hence peak SWE induce earlier melt of the snow and a rapid advance of the spring freshet detected in observations and the VIC model simulations. Increased spring and summer rainfall, however, compensates part of the precipitation decline during winter, leading to little change in annually-integrated streamflow amounts. Evapotranspiration also increases in spring and summer, further offsetting concurrent increases in precipitation and thus streamflow. Advance of the spring freshet induced by rising air temperatures is confirmed by a series of VIC model simulations forced by detrended air temperatures (Dtd-T) and precipitation (Dtd-P) over the 1949–2006 time period (see Supplementary Fig. 2). Comparing the VIC simulations driven by the forcings with trends removed from both precipitation and air temperature (Dtd-PT), from precipitation only (Dtd-P), and from air temperature only (Dtd-T) reveal that the shifts in the timing of the spring freshet and the recession to lower flows are eliminated only when daily air temperatures are detrended in the VIC model simulations. Similar features are not seen when only precipitation alone is detrended, illustrating the control of air temperature trends on streamflow timing in the Fraser River Basin.

## Discussion

This study provides new insights on the causes of streamflow timing changes observed in the FRB during the late 20th and early 21st centuries. Although total annual precipitation in the FRB has remained nearly stable over water years 1949 to 2006, changes in its type and timing coupled with warmer air temperatures have led to a significant decline in peak seasonal snowpack accumulation and an advance in the onset of melt during spring. Further to this, evapotranspiration rates during spring and summer have increased significantly, reducing runoff during the warm season despite concurrent increases in rainfall (Supplementary Fig. 2). Partitioning these results across six major sub-basins of the FRB reveals the dominance of the UF and TN sub-basins leading to these changes. This suggests that the timing of the spring freshet and the return to low summer flows of the lower Fraser River in any given year can be forecasted reasonably well by tracking conditions solely within these two upstream sub-basins. This can then provide a measure of predictability for possible spring floods generated by high snowpack levels or summer droughts when low snowpack levels are recorded^15^.

A key finding arising from this work is the VIC model’s ability to reproduce observed trends in daily streamflow for the Fraser River and six of its major sub-basins. Previous studies typically have evaluated a hydrological model’s performance in simulating observed climatological hydrographs or annual to monthly long-term trends in streamflow^16^. This effort further demonstrates the VIC model, when driven by a robust long-term meteorological forcing dataset, achieves a high degree of accuracy (e.g., NSE scores ranging from 0.47–0.97 along with other metrics; see Table 2) in simulating FRB daily streamflow trends. This is important as assessing trends in monthly streamflow could conceal changes occurring on shorter time scales. Small alpine and Arctic watersheds where snowmelt occurs rapidly causing “flashy” hydrographs could be especially susceptible to such issues^17^. This work confirms the central role land surface and hydrological models play in assessing the direction of hydrological changes (both past and future) and their driving factors, which is especially critical for the many ungauged basins of remote regions such as northern Canada^18^.

The Fraser River and its many tributaries continue to host one of the largest Pacific Ocean salmon populations^19^. Due to the FRB’s transition to a hybrid regime, its potential impacts such as lower summer flows and warmer streamflow temperatures will lead to a degradation of salmon habitat along with additional physiological stress during up-river salmon migrations. This retrospective study using the VIC hydrological model (with a focus on streamflow phase shifts in response to rapidly diminishing snowpack levels) establishes an important element for both the historical evaluations and the future predictions of the regime changes in hydroclimate variability across the FRB with potential impacts to keystone species such as salmon. Although this study suggests there are ongoing changes to the hydrological regime of the FRB, future climate change may lead to further alterations. As such, additional efforts are needed to investigate future climate scenarios and the response of the FRB hydrological system to enhanced atmospheric forcing. This will lead to the development of climate adaptation strategies^20^ for communities situated in the basin, improved water resources management, and better decision-making at all levels of governments.

## Methods

### VIC model application

To assess the FRB’s changing hydrological regime, the macroscale VIC model is employed to conduct retrospective hydrologic simulations from (water years) 1949 to 2006 6. The VIC model^21^,^22^ (along with more recent modifications) has been broadly applied to evaluate hydrological responses to a changing climate over a wide range of global river basins^6^,^23^,^24^,^25^. Driven by a forcing dataset of daily minimum and maximum air temperature, precipitation, and wind speed^26^,^27^, the VIC model is integrated on a daily time step at 0.25° spatial resolution over the entire domain of the FRB using ten 100-m elevation bands in each grid cell. VIC model outputs used in the present study include time series of daily grid-scale and the routed streamflow (m^3^ s^−1^) for the FRB’s major sub-basins following Wu *et al.*^28^ as well as evapotranspiration. Details of the VIC model implementation and application to the FRB including its forcing dataset, parameters, model outputs, calibration (performed over water years 1949–1968), and validation (performed over water years 1969–2006) are described in Kang *et al.*^6^ (see also Supplementary Figs 3 and 4). Since the model calibration is conducted by minimizing streamflow differences against the observations, the simulated streamflow for these 20 years is not independent of the observed streamflow.

Processes not considered in these VIC model simulations are changes in land cover or land use (e.g., forest harvesting or deforestation by the recent mountain pine beetle outbreak in the FRB^29^), lakes as natural reservoirs and glacier melt contributions to streamflow. Deforestation leads to less interception of precipitation by a vegetation canopy while glacier melt contributions are high in warm, dry years, offsetting low precipitation amounts^5^. As such, both these processes augment runoff productivity for the same amount of precipitation. Lakes also store water, thereby delaying streamflow generation in downstream rivers and enhancing evaporation. There is evidence of higher observed versus simulated flows in the last two decades of the study period (1986–2006) in some sub-basins (SU, NA, and CH) compared to previous decades that could indicate a growing influence of deforestation, lakes and glaciers to streamflow generation in the FRB (Supplementary Fig. 4). Such processes may be incorporated in future versions of the VIC model and applications to the FRB.

Daily streamflow trends simulated by the VIC model are assessed with corresponding observations compiled by the Water Survey of Canada. Six unregulated major sub-basins of the FRB, namely the UF, SU, NA, QU, CH and TN, are employed in this study (Table 1). Hydrometric data for the main-stem Fraser River (LF) at Hope, BC are also used in this study. This location is selected since it has the longest record of observed streamflow for the Fraser River’s main stem closest to its outlet to the Salish Sea in the Pacific Ocean, covering 94% of the basin’s drainage area.

### Statistical and trend analyses

Mean annual streamflow for the FRB’s six major sub-basins and their relative contributions to total mean annual streamflow for the Fraser River at Hope spanning water years 1949 to 2006 are first quantified. Monotonic trends in observed and simulated daily streamflow covering the period of study at each site of interest are then evaluated using the Mann-Kendall Test (MKT)^30^,^31^,^32^,^33^. The non-parametric MKT is commonly used for hydrological trend analyses as it is robust to outliers and can be applied to non-normal data^31^. Trends are considered to be statistically significant when *p* < 0.05 with a two-tailed test while slope magnitudes are extracted from the associated Kendall-Theil Robust Lines^31^. Comparisons between observed and simulated trends in daily streamflow are assessed using correlation coefficients (statistically significant when *p* < 0.05), NSE coefficients^32^, root mean square errors (RMSEs) and relative biases. Trends in observed and simulated daily streamflow for the six major FRB sub-basins are summed for comparison with those for the Fraser River at Hope. Changes in the relative contributions of the six main sub-basins to total Fraser River discharge are evaluated over 1949–2006. Grid-scale trends in daily streamflow on 1 May and 1 June are computed to establish its spatial variations across the FRB during the spring freshet. The slopes and end-points of the Kendall-Theil Robust Lines for daily streamflow are then used to re-construct the water year, daily hydrographs for the Fraser River at Hope, 1949 and 2006. This allows an exploration of the observed and simulated hydrographs for those years if the changes were entirely monotonic over the period of study as based on the MKT. A 5-day moving average is applied to the reconstructed hydrographs to remove short-term fluctuations in streamflow. The 2006 reconstructed hydrographs are also shown with a 10-day lag to better illustrate the recent advances in the onset of the spring freshets. Shifts in the timing of the spring freshet are assessed from the average number of days between the 1949 and 2006 rising limbs of the hydrograph when streamflow surpasses 2000 m^3^ s^−1^, 3000 m^3^ s^−1^ and 4000 m^3^ s^−1^, respectively^34^,^35^.

## Additional Information

**How to cite this article**: Kang, D. *et al.* Impacts of a Rapidly Declining Mountain Snowpack on Streamflow Timing in Canada’s Fraser River Basin. *Sci. Rep.* **6**, 19299; doi: 10.1038/srep19299 (2016).

## Supplementary Material

Supplementary Information

## Figures and Tables

**Table 1:** List of sub-basins of interest and the Fraser River at Hope, their abbreviations in parentheses, their mean basin elevation, gauged area, % gauged area relative to FRB at Hope, mean annual discharge (km^3^ yr^−1^), % of mean annual discharge relative to mean annual discharge for the FRB at Hope, and the 1949–2006 % change in the observed and simulated contribution of the sub-basins to mean annual discharge for the FRB at Hope.

**Sub-basin**	**Mean basin elevation [m]**	**Gauged area [km**^**2**^]	**Gauged area relative to LF [%]**	**Gauged mean annual streamflow [km**^**3**^ **yr**^**−1**^]	**Observed mean annual streamflow relative to LF [%]**	**Simulated mean annual streamflow relative to LF [%]**	**The 1949–2006 % change in the observed contribution of the sub-basins to mean annual discharge for LF**	**The 1949–2006 % change in the simulated contribution of the sub-basins to mean annual discharge for LF**
Upper Fraser (UF)	1413	32400	14.9	25.6	29.3	22.4	30.2	6.9
Stuart (SU)	1097	14200	6.5	4.0	4.8	3.3	4.7	−23.7
Nautley (NA)	1070	6030	2.8	1.0	1.1	1.0	1.1	−39.1
Quesnel (QU)	1391	11500	5.3	7.5	8.7	9.4	8.8	6.4
Chilko (CH)	1756	6940	3.2	2.8	3.2	2.0	3.3	−38.3
Thompson- Nicola (TN)	1363	54900	25.3	24.2	28.0	29.7	28.5	4.3
**Total**		125970	58.1	65.0	75.0	67.9	−	−
Fraser at Hope (LF)	1356	217000	100	87.4	100	100	0	0

**Table 2:** Nash-Sutcliffe Efficiency (NSE) scores, correlation coefficients (*r*, all statistically significant), root mean square errors (RMSE) and relative biases for the time series between observed and simulated trends in daily streamflow for all FRB sub-basins and the Fraser River at Hope, 1949–2006.

**Sub-basin**	**Upper Fraser**	**Stuart**	**Nautley**	**Quesnel**	**Chilko**	**Thompson-Nicola**	**Fraser at Hope**
NSE	0.80	0.47	0.92	0.86	0.67	0.94	0.97
*r*	0.99	0.92	0.97	0.94	0.94	0.98	0.98
RMSE (m^3^ s^−1^)	160.7	40.5	11.6	41.6	45.8	95.1	334.1
Bias (m^3^ s^−1^)	13.6	−33.5	−7.5	5.5	−26.2	−1.2	138.4
