# Supplementary material for: Impacts of a Rapidly Declining Mountain Snowpack on Streamflow Timing in Canada’s Fraser River Basin
Source: Sci Rep. 2016 Jan 27;6:19299. doi: 10.1038/srep19299 (PMC4728390; doi:10.1038/srep19299)
Supplement: Supplementary Information [file srep19299-s1.pdf]

Impacts of a Rapidly Declining Mountain Snowpack on Streamflow Timing in Canada's Fraser River Basin

Do Hyuk Kang[1,2], Huilin Gao[3], Xiaogang Shi[4], Siraj ul Islam[1] and Stephen J. Déry[1,*]

[1] Environmental Science and Engineering Program, University of Northern British Columbia, Prince George, British Columbia, Canada
[2] NASA Goddard Space Flight Center, Greenbelt, Maryland, United States of America
[3] Zachry Department of Civil Engineering, Texas A & M University, College Station, Texas, United States of America
[4] CSIRO Land and Water, Canberra, Australian Capital Territory, Australia

Supplementary Figures and Tables

Submitted in revised form to *Scientific Reports*

Manuscript Reference # SREP-14-14637-A

27 November 2015

[*]Corresponding Author: Stephen J. Déry
Environmental Science and Engineering Program
University of Northern British Columbia
3333 University Way
Prince George, BC, Canada, V2N 4Z9
E-mail: sdery@unbc.ca, Tel: (250) 960-5193, Fax: (250) 960-5845

Supplementary Information

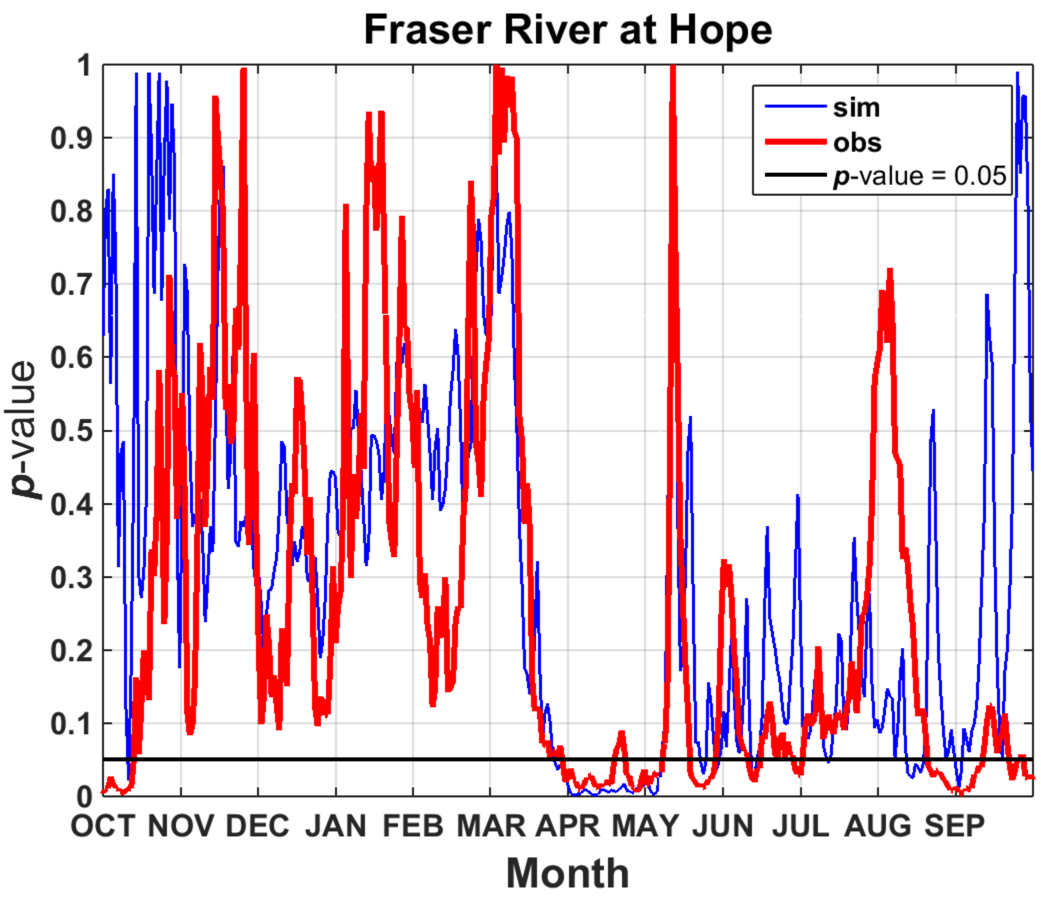

Supplementary Figure 1: Statistical significance (*p*-values) of the trend in observed (obs) and simulated (sim) daily streamflow for the Fraser River at Hope, BC, water years 1949-2006.

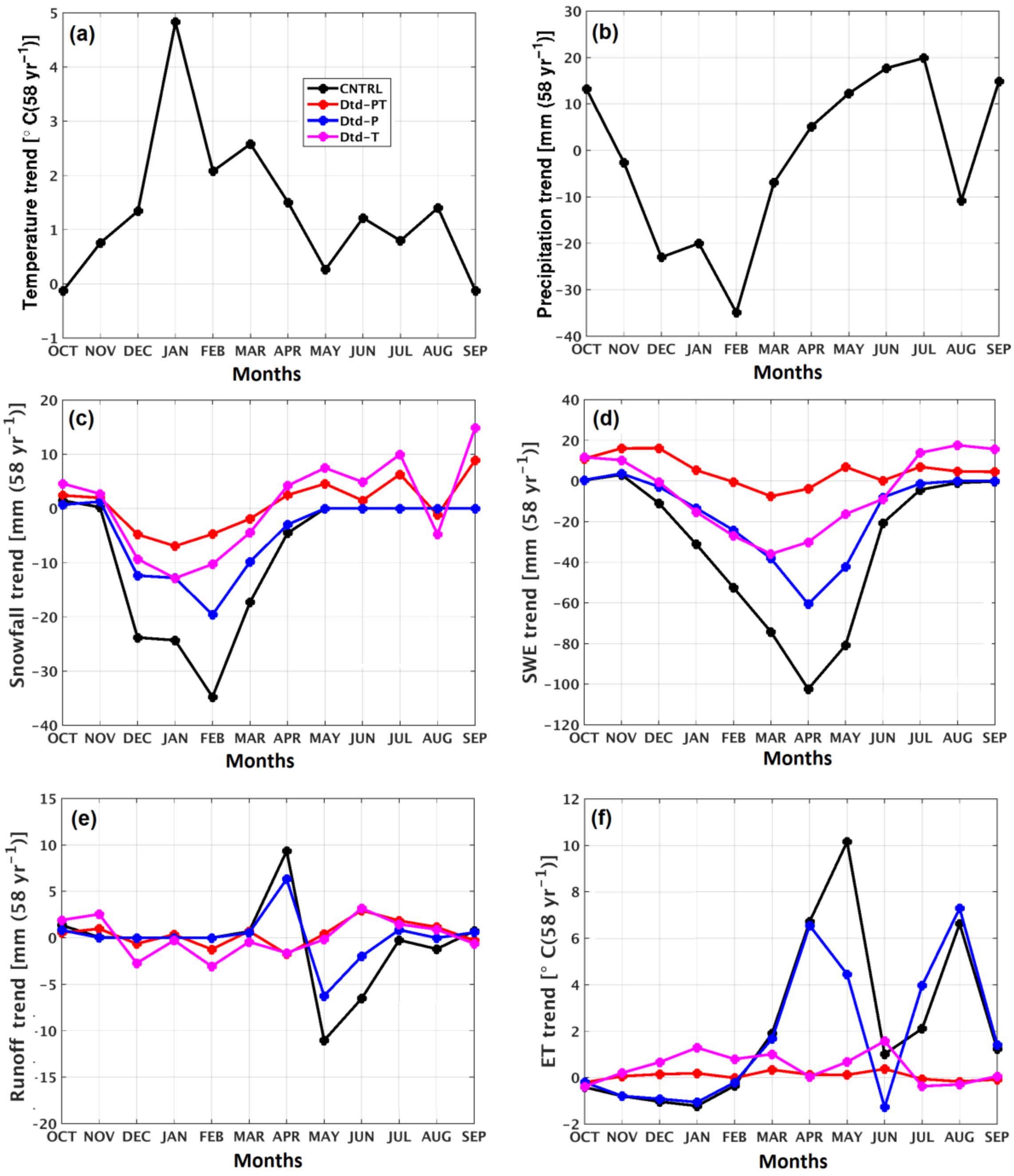

Supplementary Figure 2: Monthly trend in (a) air temperature, (b) precipitation, (c) snowfall, (d) snow water equivalent (SWE), (e) runoff and (f) evapotranspiration (ET), averaged across the FRB, water years 1949-2006. CNTRL, Dtd-PT, Dtd-P and Dtd-T represents experiments with and without removing trends from the air temperature and precipitation forcing data (see text for details).

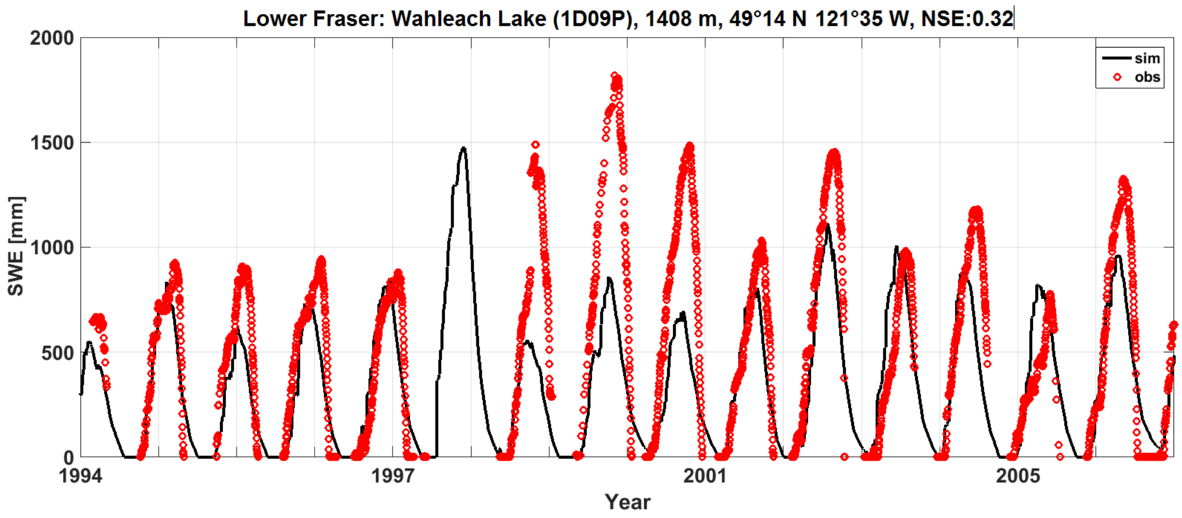

(a)

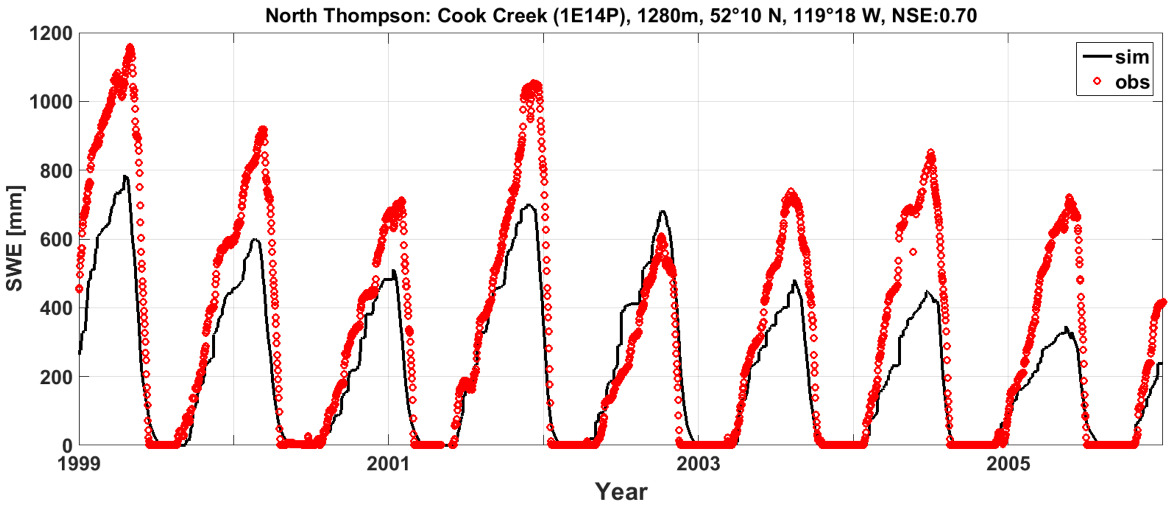

(b)

Supplementary Figure 3: Validation of daily snow water equivalent (SWE) simulations by the VIC model using BC snow pillow daily observations in the upper Fraser at (a) Wahleach Lake (ID: 1D09P) and (b) Cook Creek (ID: 1E14P).

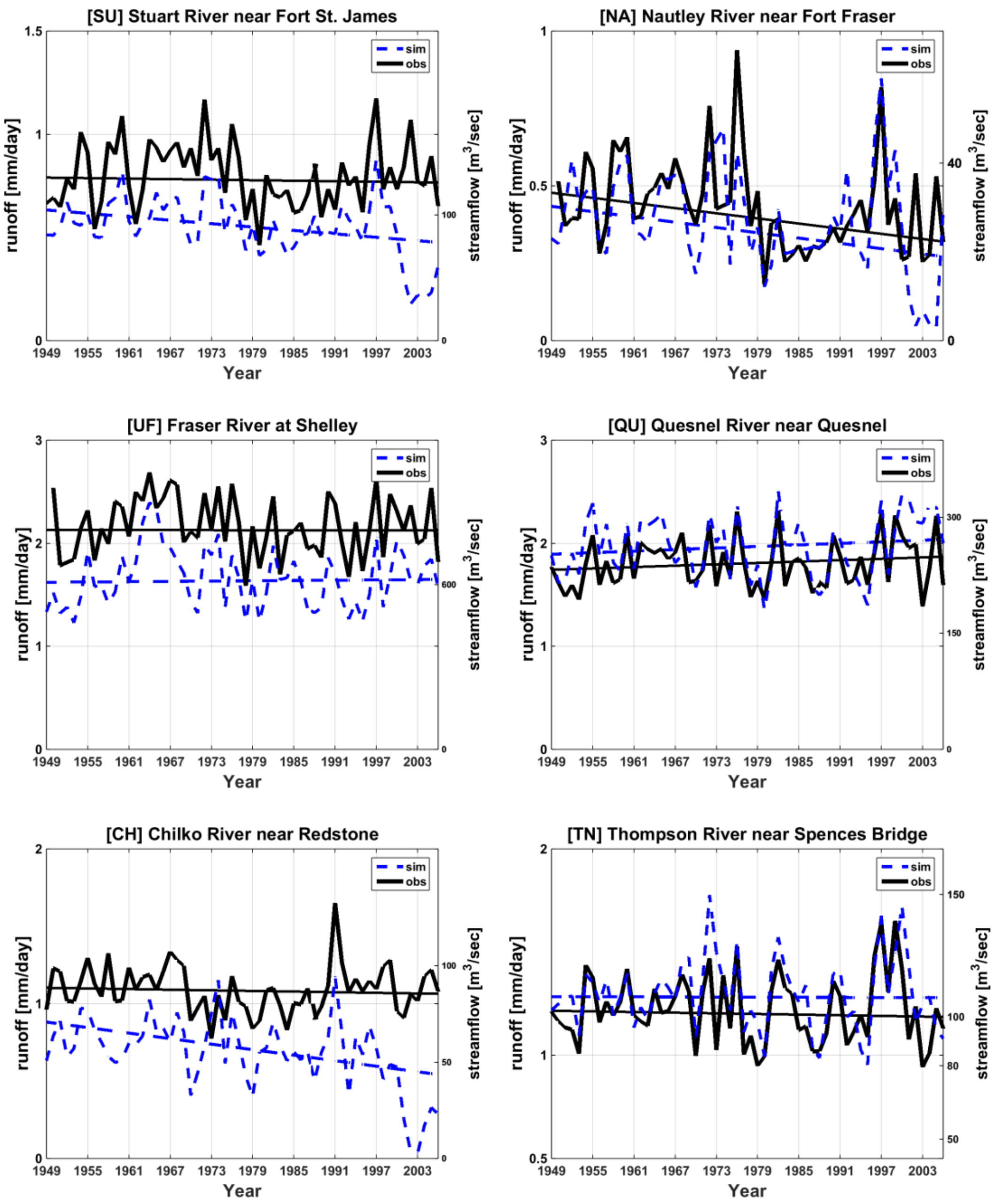

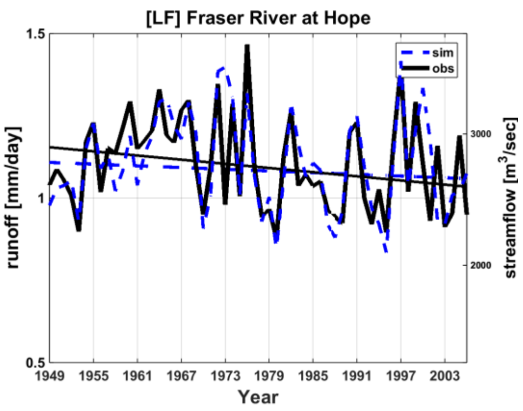

Supplementary Figure 4: Simulated (sim) and observed (obs) annual runoff for the Fraser River and its major sub-basins, water years 1949-2006. Linear trends inferred from the Mann-Kendall test are also shown.

Observed (OBS) and Simulated (SIM) Streamflow Threshold ($m^3 s^{-1}$)	Day since 1 October 1949	Day since 1 October 2006	Difference between 2006 and 1949 (days)
2000 (OBS)	202	191	-11
3000 (OBS)	211	204	-7
4000 (OBS)	223	209	-14
2000 (SIM)	199	192	-7
3000 (SIM)	209	199	-10
4000 (SIM)	215	204	-11

Supplementary Table 1: Day since 1 October 1949 and 2006 when the observed and simulated reconstructed streamflow in spring surpasses 2000, 3000 and 4000 $m^3 s^{-1}$ for the Fraser River at Hope, BC. The average 2006-1949 differences are 10.7 days and 9.3 days for the observed and simulated reconstructed hydrographs, respectively.
